# Clinical and experimental phenotype of azole-resistant *Aspergillus fumigatus* with a HapE splice site mutation: a case report

**DOI:** 10.1186/s12879-021-06279-1

**Published:** 2021-06-14

**Authors:** Yuya Ito, Takahiro Takazono, Satoru Koga, Yuichiro Nakano, Nobuyuki Ashizawa, Tatsuro Hirayama, Masato Tashiro, Tomomi Saijo, Kazuko Yamamoto, Yoshifumi Imamura, Taiga Miyazaki, Katsunori Yanagihara, Koichi Izumikawa, Hiroshi Mukae

**Affiliations:** 1 Department of Respiratory Medicine, Nagasaki University Graduate School of Biomedical Sciences, 1-7-1, Sakamoto, Nagasaki, 852-8501 Japan; 2 Department of Respiratory Medicine, Nagasaki University Hospital, 1-7-1 Sakamoto, Nagasaki, Japan; 3 Department of Infectious Diseases, Nagasaki University Graduate School of Biomedical Sciences, 1-7-1 Sakamoto, Nagasaki, 852-8501 Japan; 4 Department of Laboratory Medicine, Nagasaki University Hospital, 1-7-1, Sakamoto, Nagasaki, Japan

**Keywords:** Chronic pulmonary aspergillosis, Azole-resistant *Aspergillus fumigatus*, Virulence, HapE

## Abstract

**Background:**

The recent increase in cases of azole-resistant *Aspergillus fumigatus* (AR*Af*) infections is a major clinical concern owing to its treatment limitations. Patient-derived AR*Af* occurs after prolonged azole treatment in patients with aspergillosis and involves various *cyp51A* point mutations or non*-cyp51A* mutations. The prognosis of patients with chronic pulmonary aspergillosis (CPA) with patient-derived AR*Af* infection remains unclear. In this study, we reported the case of a patient with AR*Af* due to HapE mutation, as well as the virulence of the isolate.

**Case presentation:**

A 37-year-old male was presented with productive cough and low-grade fever. The patient was diagnosed with CPA based on the chronic course, presence of a fungus ball in the upper left lobe on chest computed tomography (CT), positivity for *Aspergillus*-precipitating antibody and denial of other diseases. The patient underwent left upper lobe and left S6 segment resection surgery because of repeated haemoptysis during voriconazole (VRC) treatment. The patient was postoperatively treated with VRC for 6 months. Since then, the patient was followed up without antifungal treatment but relapsed 4 years later, and VRC treatment was reinitiated. Although an azole-resistant isolate was isolated after VRC treatment, the patient did not show any disease progression in either respiratory symptoms or radiological findings. The AR*Af* isolated from this patient showed slow growth, decreased biomass and biofilm formation in vitro, and decreased virulence in the *Galleria mellonella* infection model compared with its parental strain. These phenotypes could be caused by the HapE splice site mutation.

**Conclusions:**

This is the first to report a case demonstrating the clinical manifestation of a CPA patient infected with AR*Af* with a HapE splice site mutation, which was consistent with the in vitro and in vivo attenuated virulence of the AR*Af* isolate. These results imply that not all the AR*Af* infections in immunocompetent patients require antifungal treatment. Further studies on the virulence of non-*cyp51A* mutations in AR*Af* are warranted.

**Supplementary Information:**

The online version contains supplementary material available at 10.1186/s12879-021-06279-1.

## Background

*Aspergillus fumigatus* is one of the most important opportunistic fungal pathogens in humans and causes aspergillosis, including invasive aspergillosis (IA), chronic pulmonary aspergillosis (CPA), and allergic bronchopulmonary aspergillosis (ABPA) [1]. Triazole antifungals are the first choice of treatment for aspergillosis [2, 3]. However, an increase in cases of azole-resistant *A. fumigatus* (AR*Af*) infections has been reported worldwide in the last 20 years, which is regarded as an emerging clinical problem due to its treatment limitations for this disease [4–7].

Infections due to azole-resistant strains are classified into the environmental and patient routes and occur through the mutation or upregulation of the *cyp51A* gene, which encodes 14-α sterol demethylase, a triazole target enzyme in *A. fumigatus* [6, 8, 9]. Environment-derived AR*Af* occurs after environmental exposure to fungicides and involves tandem repeats (TRs) in the promoter region of *cyp51A* coupled with point mutations such as TR_34_/L98H and TR_46_/Y121F/T289A, whereas patient-derived AR*Af* occurs after prolonged azole treatment in aspergillosis-infected patients and involves various *cyp51A* point mutations (G54, G138, and M220) or non*-cyp51A* mutations [9]. Although environment-derived AR*Af* infection has been associated with poor prognosis in patients with IA and CPA [7, 9–11], the prognosis of patient-derived AR*Af* infections remains unclear.

The proportion of AR*Af* exhibiting non-*cyp51A* mutations has increased over the last 5 years [12]. To date, non-*cyp51A* mutations, such as those in the *cdr1B*, *hapE,* and *hmg1* genes, have been reported to cause azole resistance [13–15]. The *cdr1B* gene encodes an ATP-binding cassette (ABC) transporter, and constitutive expression of the *cdr1B* gene leads to a decrease in intracellular drug concentration, resulting in azole resistance [13]. The *hapE* gene is a subunit of the CCAAT-binding complex (CBC), and the amino acid substitution of HapE (P88L) leads to an increase in the c*yp51A* expression and consequent azole resistance [14, 16]. Mutation in the sterol-sensing domain of the 3-hydroxy-3-methyl-glutaryl-coenzyme A reductase-encoding gene, *hmg1*, results in the accumulation of ergosterol precursors in cells, leading to triazole resistance [15].

Although some of the resistance mechanisms of AR*Af* with non-*cyp51A* mutations have been well investigated [13, 15, 17], the causes of its change in virulence remain unclear. In this study, we evaluated the clinical course of with AR*Af* due to HapE mutation, as well as the virulence of the isolate.

## Case presentation

### Case

**Fig. 1:**
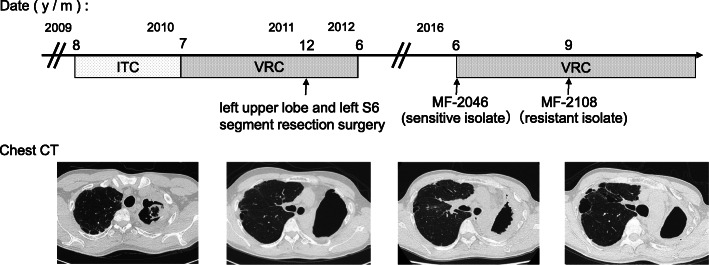
Clinical course of a patient with CPA. The patient was treated with itraconazole (ITC) from August 2009 to July 2010 and with voriconazole (VRC) until December 2011, however, he had repeated haemoptysis during treatment. He underwent left upper lobe and left S6 segment resection surgery in December 2011 and was treated with VRC for 6 months. The patient was then followed up without antifungal treatment, but relapsed in June 2016, and treatment with VRC was initiated again. The sensitive isolate, MF-2046, was obtained from sputum before treatment with VRC, while the resistant isolate, MF-2108, was obtained from sputum during treatment with VRC. Although the MF-2108 isolate was isolated from sputum in September 2016, VRC treatment was continued until November 2018, as respiratory symptoms and radiological findings improved. Since then, the patient has not been treated, but no disease progression has been observed

A 37-year-old male presented with productive cough and low-grade fever. The patient had a history of thoracic surgery for recurrent pneumothorax with chronic obstructive pulmonary disease as the underlying condition. Chest computed tomography (CT) presented a fungus ball in the upper left lobe, and the patient was positive for *Aspergillus*-precipitating antibody. Although the patient was negative for fungal culture on bronchoscopy, other diseases such as mycobacterial infection and lung cancer were excluded. The patient was diagnosed with CPA based on the chronic course, presence of a fungus ball on chest CT, and an immunological response [18]. The patient was treated with itraconazole (ITC; 400 mg/day) from August 2009 to July 2010 and subsequently, with voriconazole (VRC; 400 mg/day) until December 2011; however, he had repeated haemoptysis during treatment. The patient then underwent left upper lobe and left S6 segment resection surgery in December 2011 and was treated postoperatively with VRC (400 mg/day) for 6 months. Since then, the patient was followed up without antifungal treatment. In June 2016, he had worsening respiratory symptoms and chest CT showed thickening of the cavity wall in the upper left lobe. The sensitive isolate MF-2046 was obtained from the sputum, and VRC treatment was reinitiated due to CPA relapse. Although the resistant isolate MF-2108 was obtained from the sputum in September 2016, the VRC treatment was continued until November 2018 as respiratory symptoms and radiological findings improved. Since then, the patient has not been treated, but no disease progression has been observed (Fig. 1).

### Strains

The MF-2046 and MF-2108 isolates were obtained from the patient’s sputum in June and September of 2016, respectively (Fig. 1). Based on the macroscopic colony morphology, micromorphological characteristics, the ability to grow at 48 °C, and the sequences of the β-tubulin gene, these were identified as *A. fumigatus* sensu *strico* isolates [19, 20].

### Drug susceptibility test

In vitro susceptibility testing of the isolates was performed as previously described [19]. Briefly, minimum inhibitory concentrations (MICs) of ITC, VCZ, and amphotericin B and minimum effective concentrations (MECs) of micafungin were determined using the Clinical and Laboratory Standards Institute reference method for broth microdilution, document M38-A2, with partial modifications using a yeast-like fungus DP plate (Eiken Chemical, Tokyo, Japan) [21]. The results were evaluated according to the European Committee on Antimicrobial Susceptibility (EUCAST) clinical breakpoints.

### Short tandem repeats (STR) analysis and *cyp51A* mutation

STR analysis was performed as previously descibed [22]. Briefly, nine microsatellite regions were amplified by polymerase chain reaction (PCR) using the designed primer pairs and sequenced. The number of repeats in each region was counted from the sequences.

The *cyp51A* gene was amplified by PCR using the designed primer pairs and was sequenced. The results of the STR analysis and primers used in this study are shown in Table S1.

### Preparation of *A. fumigatus* conidia from isolates

All isolates were incubated on a potato dextrose agar (PDA) (0.4% potato starch, 2% dextrose, and 1.5% agar) (Difco Laboratories, Detroit, MI) slanted at 30 °C for 1 week. The conidia were harvested with phosphate-buffered saline (PBS) containing 1% Tween 20 solution, and the conidial suspensions obtained were passed through a sterile 40-μm strainer to remove hyphal fragments. The number of conidia was counted using a haemocytometer.

### Growth assay

A total of 1 × 10^4^ conidia /*A. fumigatus* isolate were spotted on the PDA plates and incubated for 5 days at 30 °C. Colony diameters were measured after 72, 96, and 120 h and the average diameters were calculated from three independent experiments [23].

### Biomass measurement and biofilm assay

A total of 5.0 × 10^5^ conidia / *A. fumigatus* isolate were incubated in 5 mL of yeast glucose medium at 37 °C for 24 h with shaking at 250 rpm. The precipitate obtained was then recovered by filtration, frozen at − 80 °C, lyophilized overnight, and weighed. The average biomass was calculated from three independent experiments.

Biofilm assay was performed as previously described [24, 25]. Briefly, a round-bottomed 96-well plate was inoculated with 100 μL of the conidial suspension at a density of 1 × 10^5^ conidia/mL in a Brian medium and incubated at 37 °C for 24 h. The spent culture medium was removed from each well and the adherent cells were washed three times with distilled water (dH_2_O). Next, 100 μL of 0.1% (w/v) crystal violet solution was added to each well and incubated for 10 min. This solution was carefully removed and washed twice with dH_2_O. The biofilms were destained for 10 min by adding 125 μL of 100% ethanol to each well. The absorbance of the destaining solution was measured at 595 nm.

### *Galleria mellonella* virulence assay

Healthy *G. mellonella* larvae (Oita General Service Co., Ltd., Japan) of the same size were selected for the assay. Groups of 10 larvae were inoculated with 1.0 × 10^6^ conidia into the haemocoel using a Hamilton syringe through the last left pro-leg [26]. The inoculated larvae were incubated in the dark at 37 °C and survival was monitored daily for 7 days. Ten larvae were inoculated with PBS and used as controls, and no larvae died during this time. Virulence assays were repeated three times independently.

### Whole-genome sequencing

A total of 5.0 × 10^5^ conidia / *A. fumigatus* isolate were incubated in 5 mL of yeast extract-peptone-dextrose (1% yeast extract, 2% peptone, and 2% dextrose) (Difco Laboratories, Detroit, MI, USA) broth at 37 °C for 24 h with shaking at 250 rpm. Mycelium was recovered using a sterile 40-μm strainer, rapidly frozen using absolute ethanol and dry ice, and lyophilized overnight. The lyophilized mycelium was homogenized and used for DNA extraction. DNA was extracted using a MasterPure™ DNA Purification Kit (Epicentre, Madison, WI, USA) and purified using a QIAquick PCR Purification Kit (Qiagen, Hilden, Germany) according to the manufacturer’s instructions. Whole-genome sequencing was performed by a commercial vendor (Novogene Bioinformatics Technology Co. Ltd., Beijing, China) using NovaSeq 6000 (350-bp insert library with 150-bp paired-end sequencing; Illumina, San Diego, CA).

### Sequence analysis

Adaptor sequences and low-quality reads were eliminated from paired-end sequence reads using the Cutadapt v1.16 software [27]. The sequence reads were mapped to the *Af*293 reference genome using Bowtie2 v2.3.4.1 [28, 29], and BAM files were processed with the MarkDuplicates program from Picard-tools v2.18.1 to generate analysis-ready BAM files [30]. Variants were then called to obtain multi-sample Variant Call Format file using the HaplotypeCaller program from GATK v4.1.0.0 [31]. Subsequently, the effects of the variants were predicted using the SnpEff software [32]. Single nucleotide mutations were reconfirmed using Sanger sequencing.

### RNA isolation and *cyp51A* expression

Total RNA was extracted using the RNeasy Plant Mini Kit (Qiagen, Hilden, Germany) according to the manufacturer’s instructions. Complementary DNA (cDNA) was synthesised from 2.5 μg of the total RNA using a QuantiTect Reverse Transcription kit (Qiagen, Hilden, Germany), and cDNA was used as the template for PCR using a QuantiTect SYBR Green PCR kit (Qiagen, Hilden, Germany). Quantitative reverse-transcription PCR (qRT-PCR) was performed in triplicate using a 7500 Real-Time PCR System (Applied Biosystems, Foster City, CA, USA). The mRNA abundance of the target genes was normalised to 18SrRNA. The primers used for qRT-PCR are listed in Table S1.

### Statistical analysis

Unpaired t-test was used to assess the differences in the colony diameter, and the biomass and biofilm amounts. Survival was plotted on a Kaplan–Meier curve for each strain, and log-rank (Mantel-Cox) test was used for pairwise comparison of percent survival using the GraphPad Prism 5 software (GraphPad Software, La Jolla, CA). Statistical significance was set at *P* < 0.05.

## Results

### Clinical course and characterisation of *A. fumigatus* isolates

The sensitive isolate, MF-2046, was obtained from the sputum of the patient in June 2016, 4 years after VRC and ITC treatment and surgery, before retreatment with VRC. The resistant isolate, MF-2108, was isolated from the sputum of the same patient in September 2016 during VRC treatment (Fig. 1). Microsatellite analysis showed that the two isolates obtained from the patient exhibited identical genetic backgrounds (Table 1). The MF-2046 isolate was susceptible to azole antifungal drugs (MICs: itraconazole, 0.5 mg/L and voriconazole, 0.5 mg/L), whereas the MF-2108 isolate showed resistance to azoles (MICs: itraconazole, > 8 mg/L and voriconazole, 4 mg/L). The azole-resistant isolate did not harbour a point mutation of *cyp51A* or TRs in the promoter region of *cyp51A* (Table 1).

**Table 1:** Characteristics of *Aspergillus fumigatus* isolates used in this study

Isolation date	Strain	Mirosatellite									Minimum inhibitory concentration(MIC; mg/L)			MCFG	Cyp51A
		2A	2B	2C	3A	3B	3C	4A	4B	4C	AMB	ITC	VRC	MEC(mg/L)	mutation
06/20/16	MF-2046	17	24	16	29	25	13	11	10	8	0.25	0.5	0.5	< 0.015	–
09/14/16	MF-2108	17	24	16	29	25	13	11	10	8	0.5	> 8	4	< 0.015	–

*AMB* amphotericin B, *ITC* itraconazole, *VRC* voriconazole, *MCFG* micafungin, *MEC* minimum effective concentration

Although the MF-2108 was isolated from the sputum in September 2016, VRC treatment continued until November 2018 as respiratory symptoms and radiological findings improved. Since then, the patient has not been treated, but no disease progression has been observed (Fig. 1).

### Differences in phenotype and virulence of *A. fumigatus* isolates

**Fig. 2:**
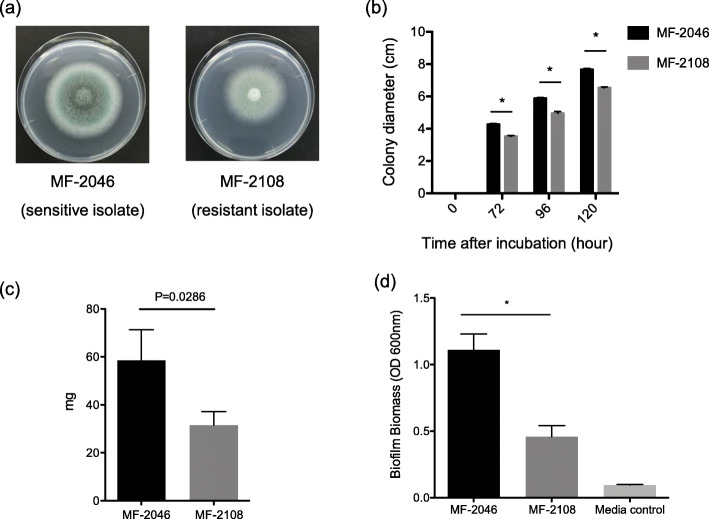
Phenotype of *Aspergillus fumigatus* isolates. **a** A total of 1 × 10^4^ conidia /*A. fumigatus* isolate were spotted on the PDA plates at 30 °C. Colony morphology was observed after 96 h incubation. **b** A total of 1 × 10^4^ conidia /*A. fumigatus* isolate were spotted on the PDA plates at 30 °C. Colony diameters were measured after 72, 96, and 120 h and average diameters were calculated from three independent experiments. Error bars represent standard deviations. *P < 0.001, Unpaired t-test. **c** A total of 5.0 × 10^5^ conidia /*A. fumigatus* isolate were incubated in 5 mL of yeast glucose (YG) media at 37 °C for 24 h with shaking at 250 rpm. The precipitates obtained were recovered by filtration, frozen at − 80 °C, lyophilized overnight, and weighed. The average biomass was calculated from three independent experiments. Error bars represent standard deviations. *P* = 0.0286, Unpaired t-test. **d** A round-bottomed 96-well plate was inoculated with 100 μL of the conidial suspension at a density of 1 × 10^5^ conidia/mL in a Brian medium and incubated at 37 °C for 24 h. The spent culture medium was removed from each well and the adherent cells were washed three times with distilled water (dH_2_O). Biofilms were stained with 100 μL of 0.1% (w/v) crystal violet solution and washed and destained with 125 μL of 100% ethanol. The absorbance of the destaining solution was measured at 595 nm. Error bars represent standard deviations. *P < 0.001, Unpaired t-test

**Fig. 3:**
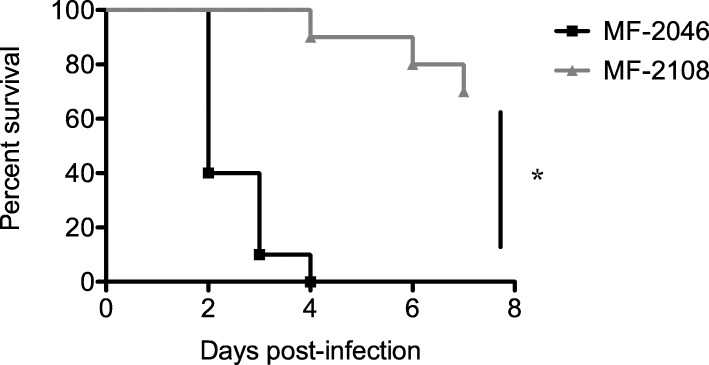
Virulence assay using the *Galleria mellonella* infection model. Groups of 10 larvae were inoculated with 1.0 × 10^6^ conidia into the haemocoel and incubated in the dark at 37 °C, and survival was monitored daily for 7 days. Kaplan–Meier curves were generated and compared using log rank (Mantel–Cox) test. *P < 0.0001

Although the colony morphologies of the two isolates were almost similar, the colony colours were different. The azole-susceptible MF-2046 isolate exhibited green colonies, whereas the azole-resistant MF-2108 isolate exhibited slightly white-in-green-coloured colonies (Fig. 2a). In the growth assay, MF-2108 colonies showed slower growth than that of the azole-susceptible MF-2046 (Fig. 2b). In addition, MF-2108 showed significantly decreased biomass and biofilm formation compared with those of MF-2046 (Fig. 2c, d). To evaluate the virulence of both isolates, a virulence assay was performed using the *G. mellonella* infection model. The MF-2108 isolate showed significantly decreased virulence compared with that of MF-2046 (Fig. 3).

### Whole-genome comparisons between *A. fumigatus* isolates

To investigate the azole-resistance mechanisms of the isolate, we compared the genome sequences of both isolates using next-generation sequencing. Compared with that in MF-2046, MF-2108 exhibited one splice site, two nonsynonymous, and two synonymous mutations (Table 2). The two nonsynonymous mutations were identified as Y622S in Afu1g09770 and F551L in Afu2g02120, which encode uncharacterised proteins and proteins exhibiting phosphopentomutase activity, respectively. Two synonymous mutations were identified in Afu2g00910 and Afu2g03450. In addition, one splice site mutation was identified in the gene encoding HapE. The mutation from G to A was observed at the last base of the third intron of the *hapE* gene. It is presumed that this mutation resulted in the intron remaining in the mature mRNA and the translation stopping at the 57th amino acid, thereby leading to the production of abnormal HapE protein (Fig. S1).

**Table 2:** Mutations detected in the azole-resistant *Aspergillus fumigatus* isolate

Gene	Description	mutation pattern
Afu1g09770	uncharacterized protein	Y622S
Afu2g00910	NB-ARC domain protein	S77S
Afu2g02120	phosphopentomutase activity	F551L
Afu2g03450	uncharacterized protein	G745G
Afu6g05300	HapE(CCAAT-binding factor complex subunit)	c.154-1G > A

### The *cyp51A* expression in the azole-resistant isolate

**Fig. 4:**
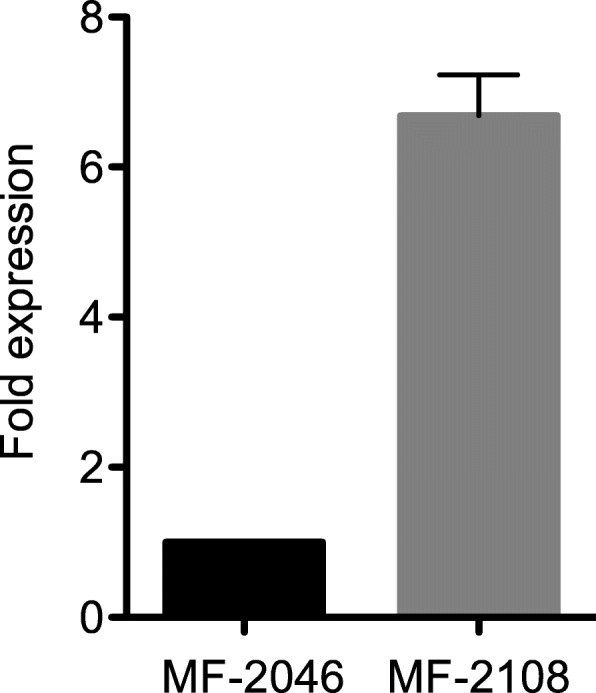
The *cyp51A* expression of *Aspergillus fumigatus* isolates. The *cyp51A* expression of MF-2108 is presented as a fold expression relative to the levels of the *cyp51A* expression in MF-2046. Error bars represent standard deviations

The *cyp51A* expression levels in both isolates were evaluated using qRT-PCR. The expression of *cyp51A* was increased by approximately six fold in the azole-resistant isolate (MF-2108) compared with that in the azole-susceptible isolate (MF-2046) (Fig. 4).

## Discussion and conclusions

To the best of our knowledge, this is the first to report a case demonstrating the clinical phenotype and virulence of an AR*Af* isolate with a HapE splice site mutation. In this study, we demonstrated that AR*Af* isolated from a patient with CPA showed slow growth, decreased biomass and biofilm formation, and decreased virulence, and these phenotypes may be caused by a HapE splice site mutation. In addition, the virulence of the isolated AR*Af* was consistent with the patient’s clinical course, and the patient did not exhibit any disease progression in respiratory symptoms or radiological findings, despite continued treatment with VRC to which the isolate with the *HapE* mutation was resistant.

A previous study reported that AR*Af* with HapE P88L substitution showed increased *cyp51A* expression levels and slow growth in an in vitro growth assay [14]. In addition, another study reported that each deletion strain of the HapB, HapC, and HapE subunits, which confer the CBC-acquired resistance to triazole antifungals, showed slow growth and decreased virulence of aspergillosis in a pulmonary and systemic mouse model [16]. Here, an AR*Af* isolate with a HapE splice site mutation showed slow growth and increased *cyp51A* expression levels, and these in vitro phenotypes were similar to those with HapE P88L mutation and a CBC mutations. These results suggest that the decreased HapE function due to the HapE splice site mutation may affect the in vitro and in vivo phenotypes, although other mutations may also affect these phenotypes. Clinically, infection with AR*Af* exhibiting a HapE P88L mutation has been reported to cause death in a patient with chronic granulomatous disease, which is caused by a primary immunodeficiency associated with phagocytic cell abnormalities, despite a clear decrease in virulence in vitro [14]*.* However, our patient did not exhibit any disease progression in either respiratory symptoms or radiological findings after infection with AR*Af* with a HapE splice site mutation. The patient had no apparent systemic immunodeficiency in this case, and these prognostic differences may have been caused by differences in host immune status.

In bacteria, long-term antibiotic exposure has been reported to cause resistance mutation, resulting in decreased growth and virulence. This evolution of a microorganism is termed the fitness costs of antibiotic resistance [33, 34]. Similarly, in fungi, fitness losses in patient-derived AR*Af* have also been frequently reported [35–37]. The *cyp51A*-mediated resistance mechanisms are not thought to be associated with fitness costs because these mutated strains do not show slow growth [36, 38]. However, in our study, the AR*Af* isolate with the HapE splice site mutation showed slow growth and decreased virulence. This adaptation of the strain due to the *HapE* mutation was considered a fitness cost.

AR*Af* infection has been suggested to be associated with poor prognosis in IA patients with attenuated immunity due to leukaemia, solid organ transplantation, and hematopoietic stem cell transplantation [39]. It has also been associated with poor prognosis in patients with CPA [11]. There are many cases of infections with AR*Af* exhibiting a *cyp51A* mutation that does not affect growth and virulence [39]. Our study results suggest that AR*Af* with non-*cyp51A* mutation may not necessarily need to be treated in patients who are not severely immunocompromised, depending on their clinical course.

Although we present the phenotype of an AR*Af* isolate with a HapE splice site mutation, our study has some limitations. First, clinically derived AR*Af* isolates have multiple mutations, and phenotypic changes are not necessarily due to a single gene change. Second, our report describes the result of the analysis of a single strain with a HapE mutation in one case.

In conclusion, this is the first to report a case demonstrating the clinical manifestation of a patient with CPA infected with an AR*Af* isolate with a HapE splice site mutation, which was consistent with the in vitro and in vivo attenuated virulence of the AR*Af* isolate. Our results imply that not all *ARAf* isolates obtained from immunocompetent patients should be considered targets for antifungal treatment. Further studies on the virulence of non-*cyp51A* mutations are warranted to better understand the resistance mechanisms in *A. fumigatus*.

## Supplementary Information

**Additional file 1: Table S1**. Primers used in this study. **Fig. S1**. HapE splice site mutation.

## Data Availability

The sequenced genome data were deposited with the DDBJ Sequence Read Archive under accession no. DRA010499. All other data and materials are included in the manuscript.

## Abbreviations

AR*Af*: Azole-resistant *Aspergillus fumigatus*
CBC: CCAAT-binding complex
cDNA: Complementary DNA
CPA: Chronic pulmonary aspergillosis
CT: Computed tomography
dH_2_O: Distilled water
IA: Invasive aspergillosis
ITC: Itraconazole
MEC: Minimum effective concentration
MIC: Minimum inhibitory concentration
PBS: Phosphate-buffered saline
PCR: Polymerase chain reaction
PDA: Potato dextrose agar
qRT-PCR: Quantitative reverse-transcription polymerase chain reaction
STR: Short tandem repeat
TR: Tandem repeat
VRC: Voriconazole
