# Correction to: Clinical and experimental phenotype of azole-resistant Aspergillus fumigatus with a HapE splice site mutation: a case report

**DOI:** 10.1186/s12879-021-06667-7

**Published:** 2021-09-16

**Authors:** Yuya Ito, Takahiro Takazono, Satoru Koga, Yuichiro Nakano, Nobuyuki Ashizawa, Tatsuro Hirayama, Masato Tashiro, Tomomi Saijo, Kazuko Yamamoto, Yoshifumi Imamura, Taiga Miyazaki, Katsunori Yanagihara, Koichi Izumikawa, Hiroshi Mukae

**Affiliations:** 1 Department of Respiratory Medicine, Nagasaki University Graduate School of Biomedical Sciences, 1-7-1, Sakamoto, Nagasaki 852-8501 Japan; 2 Department of Respiratory Medicine, Nagasaki University Hospital, 1-7-1 Sakamoto, Nagasaki, Japan; 3 Department of Infectious Diseases, Nagasaki University Graduate School of Biomedical Sciences, 1-7-1 Sakamoto, Nagasaki, 852-8501 Japan; 4 Department of Laboratory Medicine, Nagasaki University Hospital, 1-7-1, Sakamoto, Nagasaki Japan

## Correction to: BMC Infect Dis (2021) 21:573 https://doi.org/10.1186/s12879-021-06279-1

Following publication of the original article [1], the authors identified an error in Additional file 1. The correct file is given in this article.

The original article has been corrected as well.

## Supplementary Information

**Additional file 1: Table S1.** Primers used in this study. **Fig. S1.** HapE splice site mutation. The intron site is located between the 297th T and the 343rd G of the *HapE* gene. Originally, the GC-AG site is removed as an intron, but in this case, the terminal G of the intron was mutated to A. If the intron is not removed, the stop codon TAG is generated, and normal protein synthesis is not expected.
