# Supplementary material for: Clinical and experimental phenotype of azole-resistant Aspergillus fumigatus with a HapE splice site mutation: a case report
Source: BMC Infect Dis. 2021 Jun 14;21:573. doi: 10.1186/s12879-021-06279-1 (PMC8204526; doi:10.1186/s12879-021-06279-1)
Supplement: Supplementary file 1 — Additional file 1: Table S1. Primers used in this study. Fig. S1. HapE splice site mutation. [file 12879_2021_6279_MOESM1_ESM.docx]

**Clinical and experimental phenotype of patient-derived azole-resistant *Aspergillus fumigatus* with a HapE splice site mutation: a case report**

Yuya Ito^1,2^, Takahiro Takazono^1,2, *^, Satoru Koga^1,2^, Yuichiro Nakano^3^, Nobuyuki Ashizawa^2^, Tatsuro Hirayama^2^, Masato Tashiro^1,2^, Tomomi Saijo^1,2^, Kazuko Yamamoto^2^, Yoshifumi Imamura^2^, Taiga Miyazaki^2,3^, Katsunori Yanagihara^4^, Koichi Izumikawa^3^, and Hiroshi Mukae^1,2^

^1^ Department of Respiratory Medicine, Nagasaki University Graduate School of Biomedical Sciences, 1-7-1, Sakamoto, Nagasaki, Japan

^2^ Department of Respiratory Medicine, Nagasaki University Hospital, 1-7-1, Sakamoto, Nagasaki, Japan

^3^ Department of Infectious Diseases, Nagasaki University Graduate School of Biomedical Sciences, 1-7-1, Sakamoto, Nagasaki, Japan

^4^ Department of Laboratory Medicine, Nagasaki University Hospital, 1-7-1, Sakamoto, Nagasaki, Japan

*To whom correspondence should be addressed. Takahiro Takazono, Department of Respiratory Medicine, Nagasaki University Graduate School of Biomedical Sciences, 1-7-1 Sakamoto, Nagasaki 852-8501, Japan. Tel: +81 95 819 7273; Fax: +81 95 849 7285; E-mail: [takahiro-takazono@nagasaki-u.ac.jp](mailto:takahiro-takazono@nagasaki-u.ac.jp)

**Table S1. Primers used in this study**

| Primer name | Sequence（5' to 3'） |
| --- | --- |
| For cyp51A PCR amplification |  |
| Cyp51A up-F1 | GAATATATACGTCGATCTGTGTGAC |
| Cyp51A Rv | ACTATCAAAAACAGGTTTTCGCACGAGC |
| For *cyp51A* sequencing |  |
| Cyp51A up-F1 | GAATATATACGTCGATCTGTGTGAC |
| Cyp51A up-F2 | ACAGAATACTGGGCAGCGGGCTGGAG |
| Cyp51A F1 | ATGGTGCCGATGCTATGGCTTACGG |
| Cyp51A F2 | TTAGAGTCTCATGTGCCACTTATTGAGAAGG |
| Cyp51A F3 | CTCACAGCCAAAAGTCCTCGAAGAGC |
| Cyp51A F4 | TTTTCAACGTGGATGGAAAGAAAGGAGTCC |
| For *cyp51A* qRT-PCR |  |
| Cyp51A forward | TCCTGCTCCTTAGTAGCCTGGTT |
| Cyp51A reverse | GTGCTCCTTGCTTCACCTG |
| 18SrRNA forward | GGCCCTTAAATAGCCCGGT |
| 18SrRNA reverse | TGAGCCGATAGTCCCCCTAA |

**Figure legend**

**Fig. S1. HapE splice site mutation**

The intron site is located between the 297^th^ T and the 343^rd^ G of the *HapE* gene. Originally, the GC-AG site is removed as an intron, but in this case, the terminal G of the intron was mutated to A. If the intron is not removed, the stop codon TAG is generated, and normal protein synthesis is not expected.
